# Coronaviruses and SARS-COV-2

**DOI:** 10.3906/sag-2004-127

**Published:** 2020-04-21

**Authors:** Mustafa HASÖKSÜZ, Selcuk KILIÇ, Fahriye SARAÇ

**Affiliations:** 1 Department of Virology, Faculty of Veterinary Medicine, Istanbul University-Cerrahpaşa, İstanbul Turkey; 2 Microbiology Reference Lab and Biological Products Department, General Directorate of Public Health Department,Republic of Turkey Ministry of Health, Ankara Turkey; 3 Pendik Veterinary Control Institute, İstanbul Turkey

**Keywords:** Human coronaviruses, animal coronaviruses, history of coronaviruses, COVID-19, SARS-CoV-2

## Abstract

Coronaviruses (CoVs) cause a broad spectrum of diseases in domestic and wild animals, poultry, and rodents, ranging from mild to severe enteric, respiratory, and systemic disease, and also cause the common cold or pneumonia in humans. Seven coronavirus species are known to cause human infection, 4 of which, HCoV 229E, HCoV NL63, HCoV HKU1 and HCoV OC43, typically cause cold symptoms in immunocompetent individuals. The others namely SARS-CoV (severe acute respiratory syndrome coronavirus), MERS-CoV (Middle East respiratory syndrome coronavirus) were zoonotic in origin and cause severe respiratory illness and fatalities. On 31 December 2019, the existence of patients with pneumonia of an unknown aetiology was reported to WHO by the national authorities in China. This virus was officially identified by the coronavirus study group as severe acute respiratory syndrome coronavirus 2 (SARS-CoV-2), and the present outbreak of a coronavirus-associated acute respiratory disease was labelled coronavirus disease 19 (COVID-19). COVID-19’s first cases were seen in Turkey on March 10, 2020 and was number 47,029 cases and 1006 deaths after 1 month. Infections with SARS-CoV-2 are now widespread, and as of 10 April 2020, 1,727,602 cases have been confirmed in more than 210 countries, with 105,728 deaths.

## 1. Introduction

Before December 2019, 6 strains of coronavirus (CoVs) were known to infect humans and cause respiratory diseases. HCoV‐229E, HCoV‐OC43, HCoV‐NL63, and HKU1 are coronaviruses (CoVs) that normally cause only mild upper respiratory disease with rare severe infections occurring in infants, young children, and elderly people [1]. More dangerous ones are SARS‐CoV and MERS‐CoV, which can infect lower respiratory tract and trigger a severe respiratory condition in humans [2]. It is widely known that some CoVs affect birds, bats, mice, giraffe, whales, and many other wild animals, but they can also infect livestock, causing great economic loss [3,4]. Domestic animals can also play a role as intermediate hosts that enable virus transmission from the natural, wild animal hosts to humans [5,6]. In addition, domestic animals themselves can also contract bat-borne or closely related coronavirus diseases [4]. Genomic sequences that are very similar to porcine epidemic diarrhoea virus (PEDV) have been detected in bats. In 2016, a large‐scale outbreak of a disease in pigs in southern China that killed 24,000 piglets were caused by an HKU2‐related Bat-CoV, swine acute diarrhoea syndrome CoV [7,8]. This incident was the first documented case where a Bat-CoV caused a severe disease in livestock [8].

## 2. Structure of coronaviruses

**Figure 1:**
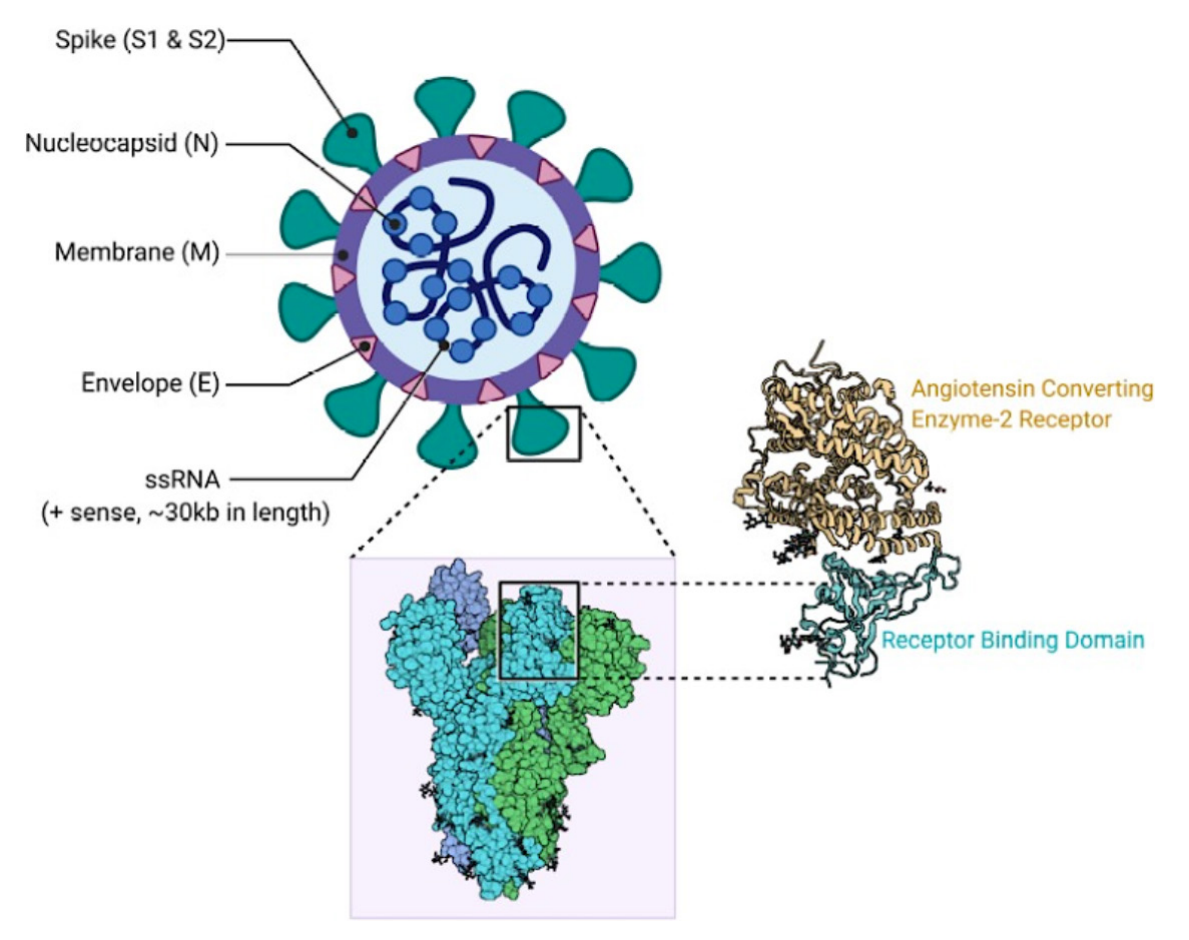
The Structure of SARS-CoV-2 virus and ACE2 protein [47]. (Contributed by Rohan Bir Singh; made with Biorender.com)

The family *Coronaviridae* is a monophyletic cluster in the order *Nidovirales* members of which are enveloped with a positive sense, single-stranded RNA genome and measures, on average, 30 kilobases [9]. *Orthocoronavirinae* subfamily contains 4 genera (Alphacoronavirus, Betacoronavirus, Gammacoronavirus, and Deltacoronavirus), and SARS-CoV and SARS-CoV-2 belong to genus betacoronavirus [10,11,12]. The coronavirus (CoV) has a single-stranded, nonsegmented RNA genome of positive polarity, and its virion contains 4 major structural proteins: the nucleocapsid (N) protein, the transmembrane (M) protein, the envelope (E) protein, and the spike (S) protein (Figure 1). However, with some coronaviruses, the full ensemble of structural proteins is not necessary for the forming of a complete, infectious virion; additional proteins may be encoded with overlapping compensatory functions [10,11,12].

The N protein is the only protein that forms the nucleocapsid and primarily functions to bind to the coronavirus RNA genome. While the N protein is involved in viral genome related processes, it plays a role in the replication of viral RNA and the host’s cellular response to viral infection. The endoplasmic reticulum localization of N protein carries a function for this in assembly and budding. Furthermore, in some coronaviruses, the N protein expression has been shown to significantly increase the production of virus-like particles [13].

Changes in the S glycoprotein are largely responsible for the host variety of coronaviruses and the variety in tissue tropism. The S glycoprotein is a type 1 membrane glycoprotein with different functional domains near the amino (S1) and carboxy (S2) termini. While the S2 subunit is a transmembrane protein mediating the fusion of viral and cellular membranes, the S1 subunit is peripheral and is associated with receptor binding functions [13,14]. Generally speaking, the S glycoprotein facilitates viral binding to susceptible cells, causes cell fusion, and induces neutralizing antibodies. Of the 2 functional subunits containing several antigenic sites, S1 and S2, the S1 monoclonal antibody appears to occur most efficiently because it has a higher level of neutralizing activity [14,15,16].

In virus assembly, the M protein of coronavirus plays a central role as it turns cellular membranes into factories where virus and host factors join to make new virus particles. The M proteins from SARS-CoV, SARS-CoV-2, MERS-CoV, MHV, FCoV, IBV, TGEV, and BCoV are targeted to the vicinity of the Golgi apparatus. Reverse genetic studies and virus-like protein (VLP) assembly studies suggest that the M protein encourages assembly by interacting with the viral ribonucleoprotein (RNP) and S glycoproteins at the budding site and by creating a network of M-M interactions capable of excluding some host membrane proteins from the viral envelope [17].

The smallest but also the most mysterious of the major structural proteins is the E protein. While the E protein is plentifully expressed inside the infected cell during the replication cycle, only a small portion is incorporated into the virion envelope [11]. Most of the protein is localized at the ER, Golgi, and ER-Golgi intermediate compartment, the site of intracellular trafficking, where it takes part in CoV assembly and budding. According to published studies, 3 roles have been proposed for the CoV E protein: a) the interaction between the cytoplasmic tails of the M and E proteins which suggests that E participates in viral assembly; b) its hydrophobic transmembrane domain is essential for the release of virions; and c) it is implicated in the virus’s pathogenesis [11,18].

Interactions between the S protein and its receptor initiate the initial attachment of the virion to the host cell. The receptor binding domains (RBD) sites within the S1 region of a coronavirus S protein vary depending on the virus; some have the RBD at the N-terminus of S1 (MHV), and others (SARS- CoV and SARS- CoV-2) have the RBD at the C-terminus of S1 [16]. To gain entry into human cells, many α coronaviruses (HCoV-229E, TGEV, PEDV, FIPV, CCoV) employ aminopeptidase N (APN) as their receptor, HCoV-NL63, SARS-CoV, and SARS-CoV-2 utilize angiotensin converting enzyme 2 (ACE2) as their receptor, MHV enters through CEACAM1, and MERS-CoV binds to dipeptidyl-peptidase 4 (DPP4) [18]. After the receptor binding, the virus must next gain access to the host cell cytosol. This is usually accomplished by acid-dependent proteolytic cleavage of the S protein by a cathepsin, TMPRRS2, or another protease, followed by fusion of the viral and cellular membranes (16).

## 3. Summary of animal coronaviruses

A wide range of animal diseases are caused by coronaviruses, and significant research on these viruses in the second half of the 20th century was triggered by their ability to cause severe disease in livestock and companion animals such as pigs, cows, chickens, dogs, and cats [19,20,21]. Transmissible gastroenteritis virus (TGEV) and PEDV, for example, cause severe gastroenteritis in young piglets that lead to significant morbidity and mortality and results in economic losses [22]. The feline infectious peritonitis virus (FIPV) results in the development of a lethal disease called feline infectious peritonitis (FIP) [23]. FIP has wet and dry forms and is similar to the human disease sarcoidosis. FIPV is macrophage tropic and may cause aberrant cytokine and/or chemokine expression and lymphocyte depletion, which results in a lethal disease [23]. The cattle industry has experienced significant losses from bovine coronaviruses (BCoV) that caused significant losses in the cattle industry, and its infection spread a variety of ruminants including elk, giraffe, deer, and camels [3,15,24]. Infectious bronchitis virus (IBV) affects the urogenital tract of chickens, causing renal disease. Egg production is significantly reduced by the infection of the reproductive tract with IBV, causing substantial industrial losses every year [20]. The most intensely studied animal coronavirus is murine hepatitis virus (MHV) which causes a variety of conditions in mice, including respiratory, enteric, hepatic, and neurologic infections. Thus, MHV is an excellent model for studying the basics of viral replication in tissue culture cells as well as for studying the pathogenesis and immune response to coronaviruses [22].

## 4. The history of coronaviruses

IBV was the first coronavirus to be reported, a virus from chickens with respiratory disease reported by Beaudette & Hudson in 1937 [20]. The murine and hepatitis viruses (MHV), another group of animal’s viruses, were first identified by Cheever at al. in 1949 [1]. In 1946, transmissible gastroenteritis in swine was first recognized. However, it was not until after the human coronaviruses (HCoVs) were discovered in the 1960s and the coronavirus genus was defined that these 3 animal diseases were found to be related [1]. An organ culture of human embryonic trachea taken from a schoolboy with a cold was described by Tyrrell & Bynoe as the first human coronavirus (B814) in 1965 [25]. When examined by an electron microscope, the virus was found to resemble avian IBV. Hamre & Procknow recovered 5 virus strains in tissue culture taken from medical students with colds around the same time [26]. Almeida & Tyrrell examined the prototype strain HCoV 229E, and its morphology was found to be identical to that of B814 and IBV [27]. Using the organ culture technique, 6 further strains were subsequently recovered including the prototype strains HCoV OC43 as well as 3 strains considered antigenically unrelated to either OC43 or 229E [1]. 

In the Guangdong province of China during the winter of 2002 to 2003, an unusual and often deadly form of pneumonia appeared, a disease subsequently labelled severe acute respiratory syndrome (SARS) [28]. This disease spread to Hong Kong in late February, and, within days, international air travel spread the virus over a wide area, seeding outbreaks in Vietnam, Singapore, Canada, and elsewhere. In July 2003, at the end of this outbreak, 8422 cases had been recorded, 916 (10.8 %) of them fatal, in 29 country across 6 continents [28]. The point of initial emergence of SARS-CoV an animal reservoir were the live animal markets in Guangdong, where diverse animal species are held, traded and sold to restaurants in response to the demand for exotic food [28]. Small mammals, such as civet cats, sold in these markets were found to harbour viruses closely related to SARS-CoV, and the initial interspecies transmission to humans probably came from these markets [29,30]. MERS-CoV was first isolated in 2012 from the lung of a 60-year-old patient who developed acute pneumonia and renal failure in Saudi Arabia. Live MERS-CoV identical to the virus found in humans was isolated from the nasal swabs of dromedary camels, further indicating that camels serve as the bona fide reservoir host of MERS-CoV. As of February 14, 2020, over 2500 laboratory confirmed cases were reported with a high case fatality of 34.4%, making MERS-CoV one of the most devastating viruses known to humans [28].

## 5. The history of COVID-19

A 41-year-old man was admitted to the Central Hospital of Wuhan on 26 December 2019, 6 days after the onset of disease. He had no history of hepatitis, tuberculosis, or diabetes and reported fever, chest tightness, an unproductive cough, pain, and weakness for 1 week on presentation. The Wuhan Centre for Disease Control and Prevention conducted an epidemiological investigation and found that the patient worked at a local indoor seafood market where, in addition to fish and shellfish, a variety of live wild animals (including hedgehogs, badgers, snakes, and birds) were available for sale as well as animal carcasses. However, no bats were available for sale, and the patient recalled no exposure to live poultry although he might have come into contact with wild animals [31]. 

On 31 December 2019, the WHO China Country Office was informed that cases of pneumonia with an unknown aetiology had been detected in Wuhan City, in the Hubei province of China [32]. From 31 December 2019 through 3 January 2020, a total of 44 patients with a pneumonia of unknown aetiology were reported to WHO by the national authorities in China. No causal agent was identified during this reporting period. Then, on 11 and 12 January 2020, WHO received further details from the National Health Commission in China that the outbreak had been associated with one of the seafood markets in Wuhan City. On 7 January 2020, the Chinese, isolated and identified a new type of coronavirus so that other countries could develop specific diagnostic kits. On 12 January 2020, China shared the genetic sequence of the novel coronavirus [34]. On 13 January 2020, the Ministry of Public Health of Thailand reported the first imported case of lab-confirmed novel coronavirus (2019-nCoV) from Wuhan, Hubei Province, China. On 15 January 2020, the Ministry of Health, Labour and Welfare of Japan (MHLW) reported an imported case of laboratory-confirmed 2019-novel coronavirus (2019-nCoV) from the same source location [35]. On 20 January 2020, the National IHR Focal Point (NFP) for the Republic of Korea reported the first case of novel coronavirus, also from Wuhan, China [36]. COVID-19’s first cases were seen in Turkey on March 10, 2020 and it was 47,029 cases and 1006 deaths after 1 month. Infections with SARS-CoV-2 are now widespread, and as of 10 April 2020, 1,727,602 cases have been confirmed in more than 210 countries, with 105,728 deaths [37].

## 6. SARS-COV-2

In an early study, phylogenetic tree showed that 2019‐nCoV (previous naming of SARS-CoV-2) significantly clustered with bat SARS-like coronavirus sequence isolated in 2015, whereas structural analysis revealed mutation in Spike glycoprotein and nucleocapsid protein. Thus, it is clear that the new 2019‐nCoV is distinct from the SARS virus which, after a mutation that gave it the ability to infect humans, was probably transmitted from bats [38]. Based on the phylogenetic tree, taxonomy, and established practice, this virus was officially identified as severe acute respiratory syndrome coronavirus 2 (SARS-CoV-2) by the Coronavirus Study Group, and the current outbreak of a coronavirus-associated acute respiratory disease was called Coronavirus Disease-19 (COVID-19) [39].

Human epidemics with variable clinical severity featuring respiratory and extra-respiratory manifestations have been caused by a number of CoVs: SARS-CoV, SARS-CoV-2, and MERS-CoV (betaCoVs of the B and C lineage, respectively) [40]. With SARS-CoV and MERS-CoV, mortality rates have been seen up to 10% and 35%, respectively which places SARS-CoV-2 in the betaCoVs category [41]. This variety has a round or elliptic, and often pleomorphic form and a diameter of approximately 60–140 nm. Like other CoVs, it is sensitive to ultraviolet rays and heat [42]. Another effective means of inactivation is by lipid solvents including ether (75%), ethanol, chlorine-containing disinfectant, peroxyacetic acid, and chloroform except for chlorhexidine [43,44].

SARS-CoV-2 and SARS-CoV-1 have similar stability. Both viruses could be detected in aerosols up to 3 h post-aerosolization, up to 4 h on copper, up to 24 h on cardboard and up to 2 to 3 days on plastic and stainless steel. In aerosols, SARS-CoV-2, and SARS-CoV-1 exhibited similar half-lives with median estimates of around 2.7 h [43]. With the median half-life estimate for SARS-CoV-2 being around 13 h on stainless steel and around 16 h on polypropylene, both viruses exhibit relatively long viability on these surfaces compared to copper or cardboard [44].

**Figure 2:**
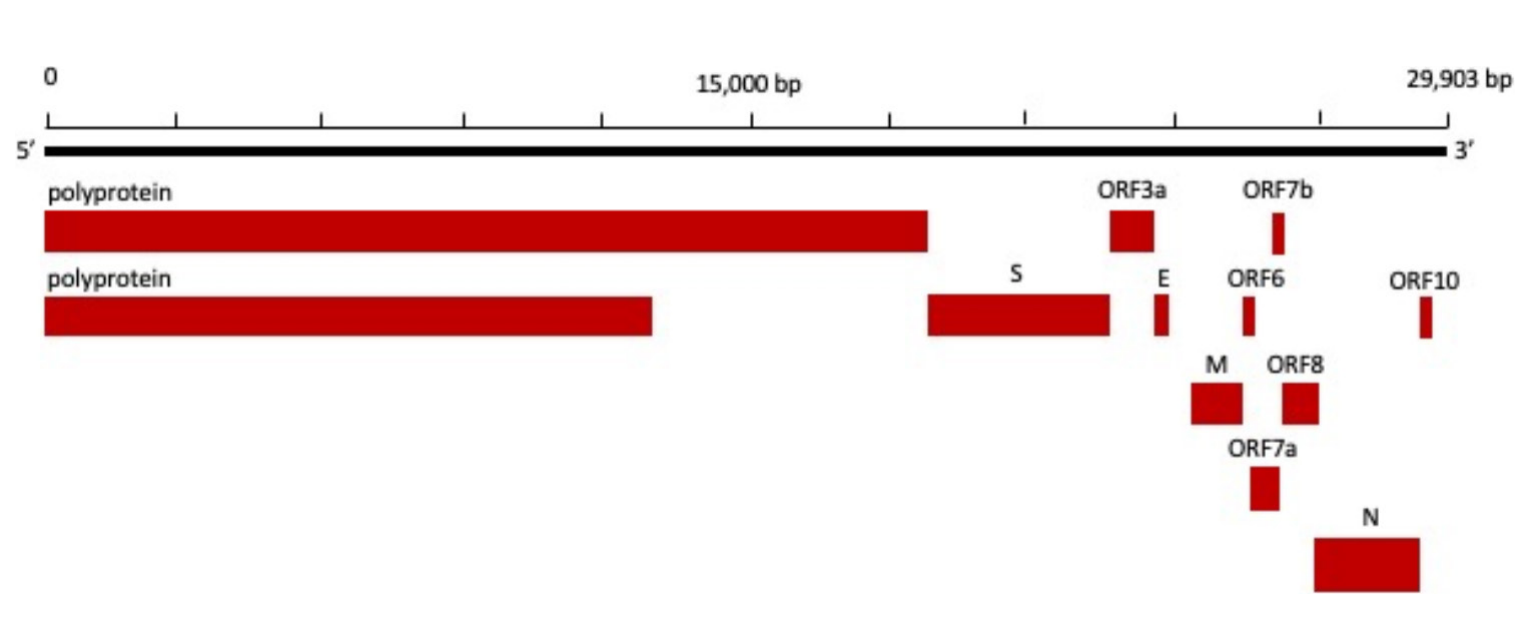
Genomic structure of SARS-CoV-2 Wuhan Hu-1 strain. Accession: NC_045512 [9]

Chan et al. [45] have shown that, in genetic terms, the genome of the new HCoV, isolated from a cluster-patient who presented with atypical pneumonia after visiting Wuhan, had a 89% nucleotide identity with bat SARS-like-CoVZXC21 and 82% with that of human SARS-CoV [45]. For this reason, the new virus was called SARS-CoV-2, its single-stranded RNA genome containing 29,903 nucleotides encoding for 9860 amino acids (Figure 2) [9]. While its origins are not completely understood, these genomic analyses suggest that SARS-CoV-2 probably evolved from a strain found in bats. However, the possible intermediate between bats and humans, the potential amplifying mammalian is not known. It is not even certain that this intermediary exists since the virulence toward humans could have been directly triggered by the mutation in the original strain [46]. 

While it is believed that bats and palm civets were the natural and intermediate reservoirs for SARS-CoV, respectively, it has been isolated from animals and adapted to lab cell culture [46,47]. It is also believed that SARS-CoV was transmitted from palm civets to humans in an animal market in southern China, and while the animal source of the outbreak is currently unknown, SARS-CoV-2 also reportedly infected humans in an animal market in Wuhan. Critically, the capacity for SARS-CoV-2 to transmit from human to human has been confirmed [48]. An envelope-anchored spike protein mediates coronavirus entry into host cells by first binding to a host receptor and then fusing viral and host membranes [49].

When the SARS-CoV-2 virus was compared with S gene of SARS-CoV, bat-CoV (As6526), bat-CoV (RaTG13), mink-CoV, and pangolin-CoV, it was found 71.41%, 68.17%, 92,86%, 30,89%, and 90% similarity, respectively [50]. When the homology of SARS-CoV-2 and these coronaviruses is less than 75%, it would be presumed that SARS-CoV-2 is not the same virus like the coronaviruses arising from these wild animals [50]. It is also clear from these results that the 2 viruses, SARS-CoV-2 and bat coronavirus RaTG13, are closely related [50]. It was also reported that the SARS-CoV-2 virus did not come directly from pangolins (24), so the relationship between SARS-CoV-2 and pangolin coronavirus and whether the pangolin is the intermediate host of SARS-CoV-2 requires further investigation [51,52].

**Figure 3:**
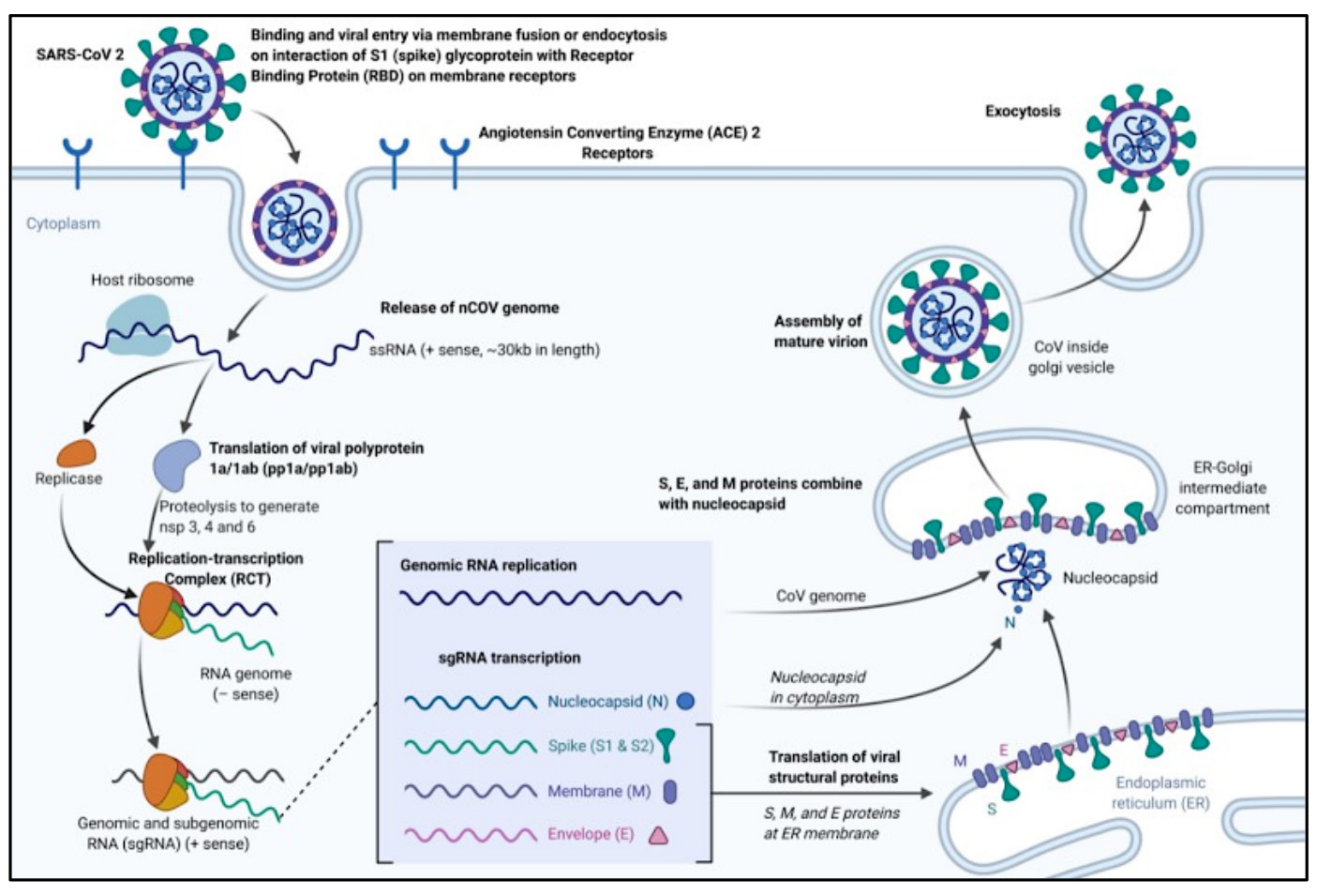
Binding, viral entry, and replication cycle of SARS-CoV-2 [47]. (Contributed by Rohan Bir Singh, made with Biorender.com).

Spike protein contains 2 subunits, S1 and S2 [53]. S1 contains a receptor binding domain (RBD), which is responsible for recognizing and binding with the cell surface receptor. S2 subunit is the “stem” of the structure, which contains other basic elements needed for the membrane fusion [53]. The spike protein is the common target for neutralizing antibodies and vaccines. It has been reported that COVID-19 can infect the human respiratory epithelial cells through interaction with the human ACE2 receptor (Figure 3). Indeed, the recombinant Spike protein can bind with recombinant ACE2 protein [54,55]. The ACE2 protein is reportedly present in type 1 and type 2 pneumocytes, enterocytes of all parts of the small intestine, the brush border of the proximal arteries, and veins of all tissues studied, and arterial smooth muscle cells [54]. This localization of ACE2 explains the tissue tropism of SARS-CoV for the lung, small intestine, and kidney. However, notable discrepancies include virus replication in colonic epithelium, which has no ACE2, and no virus infection in endothelial cells, which have ACE2, other receptors, or co-receptors such as LSIG that explain such discrepancies [54].

The pathophysiology and virulence mechanisms of CoVs– and thus of SARS-CoV-2–have links to the function of the nonstructural protein (nsps) and structural proteins. For instance, a nsp can block the host innate immune response of the host, according to the research. It is known that the envelope, among the functions of structural proteins, plays a crucial role in virus pathogenicity because it promotes viral assembly and release, but many features (e.g., those of nsp 2 and 11) have not been described yet [56].

Infectious disease experts and multiple international and domestic human and animal health organizations (CDC, OIE, and WHO) agree that there is no evidence at this point indicating that pets can spread COVID-19 to other animals, including people. Although there has not been reports of pets becoming sick with COVID-19, out of an abundance of caution, it is recommended that those of ill with COVID-19 should limit contact with animals until more information is known about the virus. On the other hand, Shi et al. [2020] report that cats and ferrets can be experimentally infected with SARS-CoV-2, but not dogs, pig, chickens, and ducks. In this study, which is the only one in this subject, they claim that cats can spread SARS-CoV-2 by respiratory and other cats can be infected [57].

**Figure 4:**
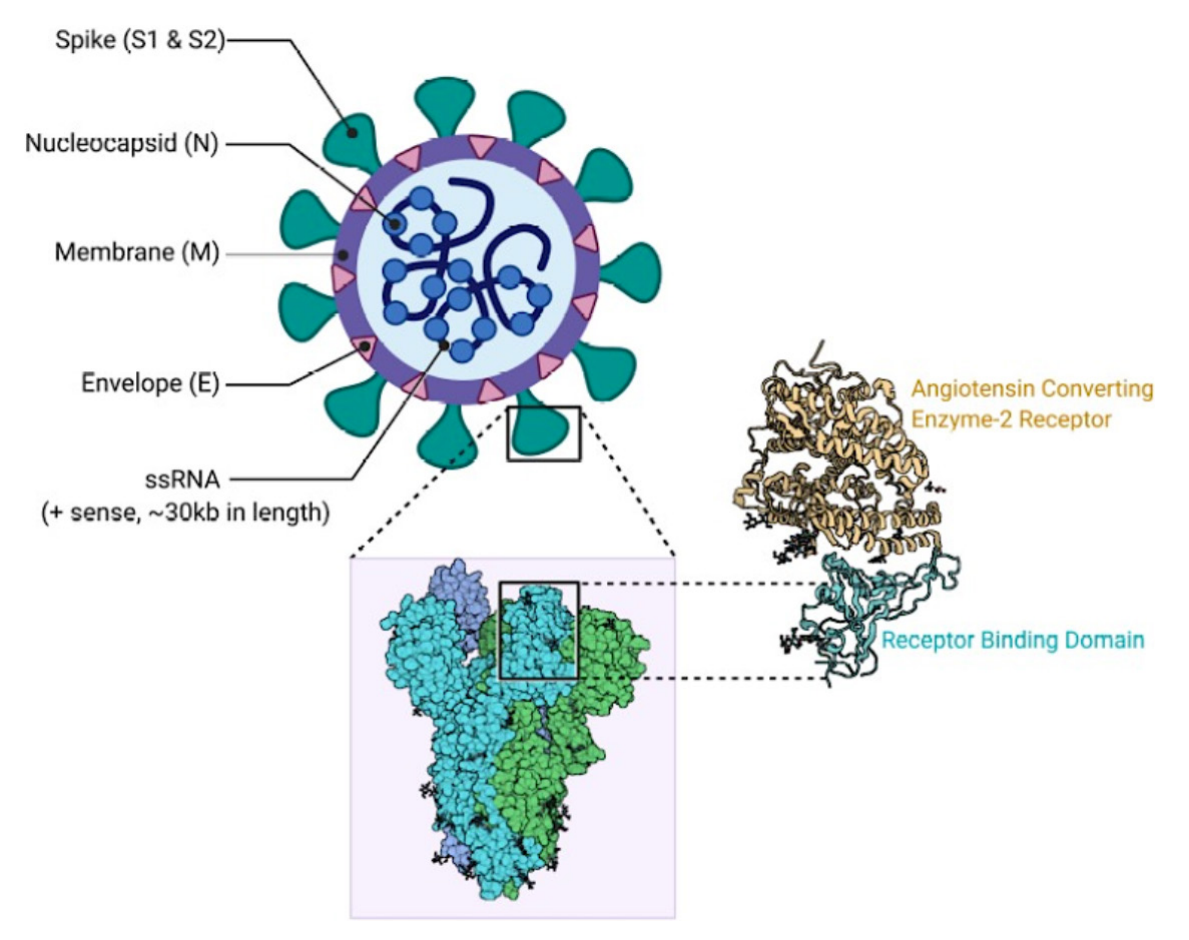
Animal origins of human coronaviruses [5].

Ultimately, novel coronaviruses are likely to emerge periodically in humans because of frequent cross-species infections and occasional spillover events (Figure 4), given the high prevalence and wide distribution of coronaviruses, the large genetic diversity, and frequent recombination of their genomes, and an increasing level of human-animal interaction.

## Acknowledgments

Mustafa Hasöksüz and Selçuk Kılıç are the members of COVID-19 Advisory Committee of Ministry of Health of Turkey.
